# Integrating biology, frailty, and patient values: The 5M framework for older adults with multiple myeloma

**DOI:** 10.46989/001c.171785

**Published:** 2026-11-03

**Authors:** Atsushi Isoda

**Affiliations:** 1 Department of Hematology, Iryohojin Hoshiiin, Maebashi, Gunma, Japan

**Keywords:** Multiple myeloma, Frailty, Geriatric oncology, Multidisciplinary care, Patient-centered decision-making

## Abstract

Multiple myeloma (MM) predominantly affects older adults, in whom frailty, comorbidities, and psychosocial factors complicate treatment decision-making. Existing tools, including the International Myeloma Working Group (IMWG) frailty score and comprehensive geriatric assessment (CGA), provide valuable insights but do not offer a structured approach that integrates disease biology, treatment feasibility, and patient values. To address this gap, we developed the 5M Framework for Older Adults with Multiple Myeloma (5M-OMM), a five-domain model that supports stepwise, multidisciplinary decision-making. The framework first defines a biologically informed strategy aligned with patient priorities and then incorporates functional, psychological, and social factors to refine the treatment plan. Application to representative clinical cases illustrates how selecting initial therapy according to disease biology and patient priorities, followed by adaptation to frailty and comorbidity burden, can improve feasibility and reduce the risk of undertreatment when frailty tools are used in isolation. The 5M-OMM complements existing geriatric oncology approaches by providing a practical structure for reassessment and multidisciplinary shared decision-making in older adults with MM.

## 1. Introduction

Multiple myeloma (MM) is primarily a disease of older adults, with a median age at diagnosis of approximately 70 years.^1,2^ Over the past two decades, therapeutic advances—including proteasome inhibitors, immunomodulatory drugs, monoclonal antibodies, and, more recently, T-cell–redirecting therapies (chimeric antigen receptor [CAR] T-cell therapy and bispecific antibodies)—have improved survival outcomes.^3–6^ Nevertheless, many older adults experience early mortality or discontinue treatment, often due to treatment-related toxicities.^7–9^ Treatment selection therefore remains challenging, given substantial heterogeneity in physiologic reserve, multi-morbidity, cognitive function, and social support.^10^

In parallel, deeper responses—including complete remission and minimal residual disease (MRD) negativity—have become increasingly achievable, even among older adults with MM.^3,4,11–13^ However, treatment goals vary widely in this population. Some patients prioritize preservation of independence and quality of life and may thus prefer less intensive regimens.^14–16^ Cognitive impairment, communication barriers, and implicit or explicit biases may further complicate shared decision-making, underscoring the need for structured approaches to goal-concordant treatment planning.^17–19^

Existing guidelines from the International Myeloma Working Group (IMWG) and the National Comprehensive Cancer Network (NCCN) provide essential recommendations, but they do not fully capture the complexity of treatment selection in older adults.^20,21^ In practice, clinicians must balance biological risk—defined by disease aggressiveness, cytogenetic abnormalities, and organ-threatening involvement—against feasibility constraints imposed by frailty, comorbidities, and available caregiver and health system support.^10,22^ This tension is often accentuated in settings where access to novel agents or supportive care resources is limited.^23^

Frailty assessment tools—including the IMWG frailty score and other validated instruments—offer prognostic information beyond chronological age.^7,24–27^ Simple objective measures of physical function, such as gait speed, also predict survival in older adults with hematologic malignancies, including MM.^28^ Frailty is increasingly recognized as a dynamic construct that can evolve during therapy,^29^ and its trajectory may vary substantially across the disease course in older adults with MM.^29,30^ Clinical trials of frailty-adapted strategies further suggest that adjusting treatment intensity according to frailty status can improve tolerability without compromising efficacy.^31,32^ Together, these data support individualized, longitudinal assessment, particularly in octogenarians (≥80 years) and patients with multi-morbidity.^33,34^

Beyond frailty tools, comprehensive geriatric assessment (CGA) provides a multidimensional evaluation of functional, cognitive, psychological, and social domains and can identify vulnerabilities not captured by performance status alone.^35^ CGA is feasible in older adults with newly diagnosed MM, and nutritional status, depressive symptoms, and comorbidity burden are associated with overall survival.^36^ However, implementation of frailty tools and CGA remains variable in routine practice, and results are not consistently incorporated into a stepwise process that guides actionable treatment decisions. These limitations underscore the need for a unified, clinically applicable framework that integrates biological risk, practical feasibility, and patient values within a structured decision-making sequence.^37–39^

To address this need, we developed the 5M Framework for Older Adults with Multiple Myeloma (5M-OMM), which organizes clinical decision-making across five domains: Myeloma Status/Matters Most, Medications/Modalities, Mobility/Multi-morbidity, Mind (Mental)/Support Members, and Menu/Motion (Fig. 1). The 5M-OMM is not a rigid algorithm; rather, it provides a sequential, adaptable structure that anchors decisions in disease biology and patient priorities while incorporating feasibility assessment. Through structured multidisciplinary discussion and iterative reassessment, the framework supports individualized treatment planning for older adults with MM.

**Figure 1.**
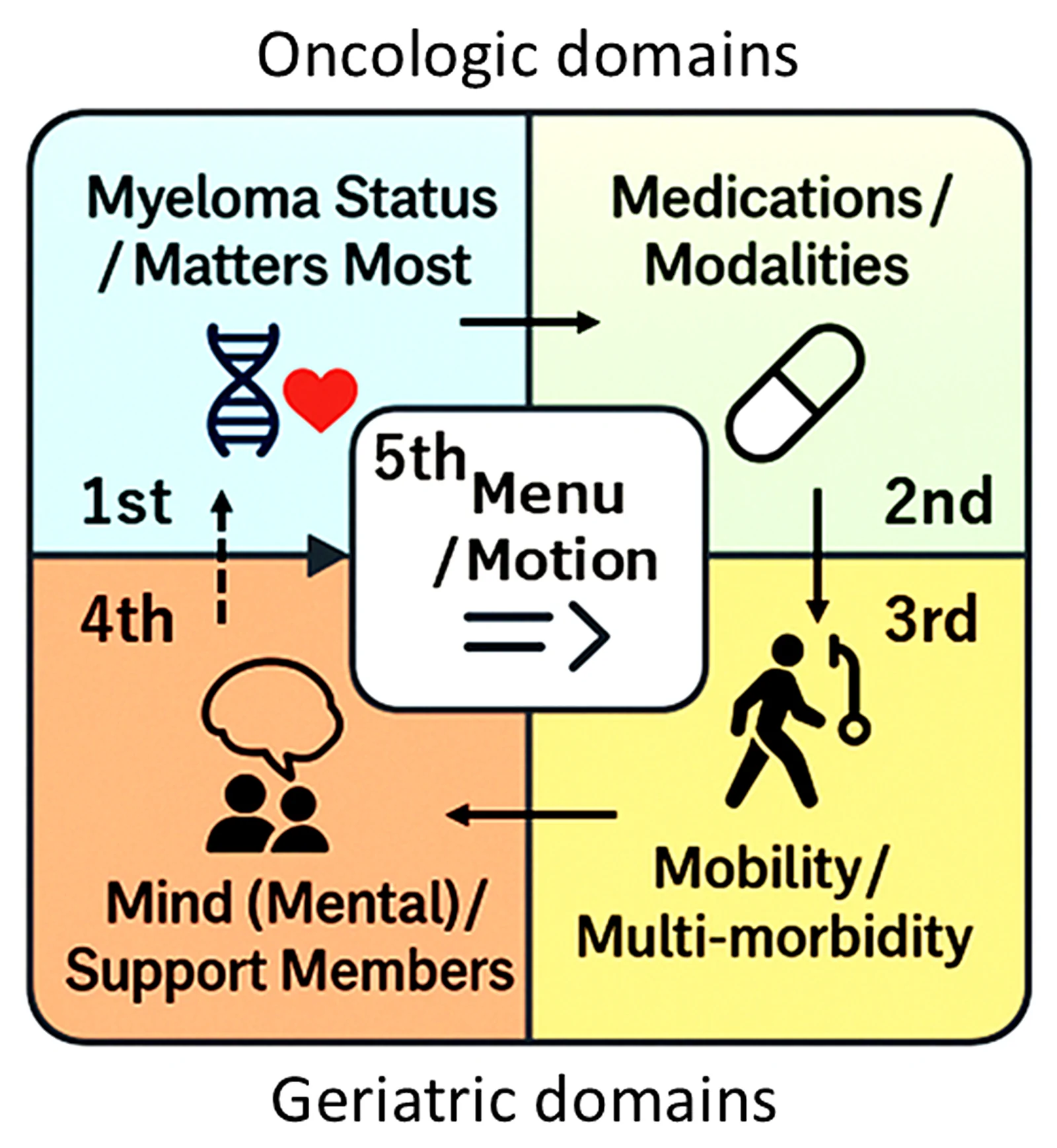
The 5M Framework for Older Adults with Multiple Myeloma (5M-OMM). The framework comprises four sequential domains arranged in quadrants: Myeloma Status/Matters Most (1st), Medications/Modalities (2nd), Mobility/Multi-morbidity (3rd), and Mind (Mental)/Support Members (4th). The central 5th M, Menu/Motion, integrates these inputs into an implementable treatment and supportive care plan. Solid arrows indicate the stepwise flow from defining biological risk and patient priorities to selecting therapeutic options and assessing feasibility across functional, medical, cognitive, and social domains. The dashed arrow denotes iterative re-looping and reassessment as feasibility evolves over time.

## 2. Methods

### 2.1. Theoretical Foundation

The 5M-OMM draws on concepts from geriatric oncology, shared decision-making, and multidisciplinary team (MDT) care. It aligns treatment intensity with physiologic reserve, incorporates patient-defined goals into treatment planning, and structures MDT discussions of multi-morbidity and psychosocial needs. The framework also applies sequential clinical reasoning with iterative reassessment. Collectively, these principles inform an integrated approach that complements established tools, including the IMWG frailty score and CGA, while providing a stepwise structure for clinical decision-making.

### 2.2. Development of the Framework

The 5M-OMM was developed between 2020 and 2024 through an iterative, clinically grounded process across two hematology centers. Feedback was collected from a multidisciplinary core group of 12–15 clinicians—including hematologists, geriatricians, nurses, pharmacists, rehabilitation therapists, and social workers—through recurrent multidisciplinary case conferences and routine clinical encounters. Across successive iterations, this feedback was synthesized to (1) define the sequencing of the five domains, (2) clarify the integration of oncologic and geriatric considerations, and (3) refine how feasibility assessment informs treatment planning.

### 2.3. Structure of the 5M-OMM

Fig. 1 summarizes the 5M-OMM framework, which comprises five domains:

1st M: Myeloma Status/Matters Most — Define biological risk (e.g., cytogenetics, disease stage, and CRAB features) and clarify patient values and priorities.

2nd M: Medications/Modalities — Select therapeutic options based on clinical evidence, eligibility, and treatment objectives.

3rd M: Mobility/Multi-morbidity — Evaluate physical function (e.g., mobility, activities of daily living [ADL], instrumental activities of daily living [IADL], and fall risk) and comorbidity burden (e.g., cardiac, renal, respiratory, and metabolic conditions).

4th M: Mind (Mental)/Support Members — Assess psychological well-being, cognition, motivation, caregiver support, and social resources.

5th M: Menu/Motion — Translate these inputs into a personalized treatment and supportive care plan and coordinate its delivery.

Table 1 outlines the core questions and clinical focus for each domain; Supplementary Table S1 provides operational examples to support application.

**Table 1.** Conceptual Summary of Each Domain in the 5M Framework for Older Adults with Multiple Myeloma (5M-OMM)

**Domain**	**Core Question**	**Clinical Focus**
1st M: Myeloma Status/Matters Most	What is the biological imperative, and what matters most to the patient?	Disease biology, stage, symptom burden, prior treatment context, organ-threatening involvement, and overarching goals and values.
2nd M: Medications/Modalities	Which options are medically appropriate and aligned with patient priorities?	Evidence-based regimen selection considering efficacy, toxicity, complexity, treatment burden, and patient preferences.
3rd M: Mobility/Multi-morbidity	What limits or enables safe treatment delivery?	Frailty, functional reserve (mobility, ADL/IADL, fall risk), nutrition, comorbidity burden, organ reserve, and polypharmacy.
4th M: Mind (Mental)/Support Members	What psychosocial and cognitive factors influence safety and adherence?	Cognition, mood, adherence capacity/self-management ability, caregiver availability, and social support/access barriers.
5th M: Menu/Motion	What is the individualized, feasible, multidisciplinary plan?	Treatment and supportive care plan; role assignment; monitoring and follow-up; triggers for reassessment and treatment modification.

Abbreviations: ADL, activities of daily living; IADL, instrumental activities of daily living.

### 2.4. Application Process

The 5M-OMM is applied through a structured four-step approach:

(1) Information gathering: Team members compile patient-specific information across the 1st–4th Ms—including disease biology, candidate treatment options, functional status, psychosocial context, and patient priorities—and organize it within the corresponding domains.

(2) Structured discussion: The discussion begins with the 1st M to define biological risk and clarify patient priorities, then proceeds to the 2nd M to review evidence-based options (the “oncologic domains,” 1st–2nd Ms). The team next evaluates practical feasibility across the 3rd and 4th Ms—function, comorbidities, cognition, psychological well-being, and social support (the “geriatric domains,” 3rd–4th Ms)—and returns to the 2nd M as needed to revise the regimen. Alignment with patient priorities is reaffirmed before proceeding to the 5th M.

(3) Translation into the 5th M: The team synthesizes these considerations into a feasible, prioritized care plan, specifying the regimen, supportive measures, team roles, and follow-up schedule.

(4) Implementation and reassessment: The plan is implemented and refined through serial reassessment as clinical status and feasibility evolve.

### 2.5. Anticipated Benefits

The 5M-OMM offers several anticipated advantages:

(1) Comprehensive integration: Integrates biological, functional, psychosocial, and patient-priority information into a single-page summary that supports at-a-glance review and decision-making.

(2) Multidisciplinary collaboration: Facilitates structured, inclusive multidisciplinary discussion and shared ownership of feasible, goal-concordant care.

(3) Clarity and convergence: Provides an explicit, stepwise sequence that supports transparent decision-making and convergence on a coordinated care plan.

(4) Longitudinal consistency: Embeds iterative reassessment as clinical status and feasibility evolve over time, enabling timely refinement of the plan.

(5) Transferable learning: Supports cross-case learning by carrying forward key insights from one patient’s assessment to inform subsequent care.

## 3. Results

The following cases illustrate how the 5M-OMM enables structured, sequential decision-making in older adults with MM. Additional clinical details are provided in the Supplementary Appendix.

### 3.1. Case 1 — Newly diagnosed MM in an octogenarian: aligning biological appropriateness with home-based care

Case summary: A frail octogenarian prioritized longevity and home-based therapy; biologically appropriate treatment was made feasible through tailored supportive care and social interventions.

An 83-year-old man presented with a vertebral compression fracture and was diagnosed with revised International Staging System (R-ISS) stage II MM. He had an ECOG performance status of 3, and cytogenetic testing showed no high-risk abnormalities. His IMWG frailty score was 4 (age, 2; ADL, 1; IADL, 1; comorbidity, 0), classifying him as frail. He prioritized longevity, pain control, and the ability to continue treatment at home.

Based on the 1st M (Myeloma Status/Matters Most), the team clarified biological risk and patient priorities. The 2nd M (Medications/Modalities) then guided selection of daratumumab, lenalidomide, and dexamethasone (DRd) as an evidence-based regimen aligned with these goals. Supportive care included early corticosteroid tapering, antiviral prophylaxis, and updated immunizations. In the 3rd M (Mobility/Multi-morbidity), feasibility was optimized by initiating inpatient rehabilitation to enhance strength and ambulation and by adjusting his type 2 diabetes regimen. In the 4th M (Mind [Mental]/Support Members), mild memory impairment was addressed by involving his daughter in medication management and advance care planning. After one month, with home care and visiting nursing in place, he transitioned safely to home-based therapy.

Framework insight: Establishing biological risk and patient priorities upfront defined an appropriate therapeutic direction, while targeted adaptations in the 3rd and 4th Ms enabled safe treatment delivery at home. Without this structured integration, frailty could have led to premature de-escalation and undertreatment.

### 3.2. Case 2 — Relapsed MM with a strong preference for outpatient continuity: preserving caregiving responsibilities

Case summary: A fit older adult prioritized outpatient-based care to maintain caregiving responsibilities; early clarification of values informed regimen selection and visit logistics.

A 74-year-old woman with biochemical relapse (R-ISS stage II, without 1q gain or extramedullary disease) prioritized outpatient-based care to preserve caregiving responsibilities for a grandchild. She had previously received bortezomib, lenalidomide, and dexamethasone (VRd) induction followed by lenalidomide maintenance. Her IMWG frailty score was 0, classifying her as fit.

Based on the 1st M (Myeloma Status/Matters Most), the team characterized the relapse as slowly progressive and confirmed that outpatient continuity was central to her goals. The 2nd M (Medications/Modalities) then guided selection of daratumumab, pomalidomide, and dexamethasone (DPd), given preserved organ function and demonstrated adherence. In the 3rd and 4th Ms (Mobility/Multi-morbidity and Mind [Mental]/Support Members), feasibility was optimized by tailoring visit scheduling to transportation constraints and caregiver availability. Her strong self-management and consistent family support helped maintain outpatient continuity.

Framework insight: Clarifying Matters Most upfront ensured alignment between treatment planning and her primary caregiving role. By allowing patient-defined goals to inform regimen selection early, the 5M-OMM reduced the likelihood of escalation to more burdensome care that could have disrupted outpatient continuity.

### 3.3. Case 3 — Newly diagnosed high-risk MM: enabling stepwise intensification through adaptive sequencing

Case summary: High-risk features created an urgent need for rapid cytoreduction, but initial feasibility constraints prompted a safer induction strategy with planned escalation as renal function and mobility improved.

A 77-year-old man presented with high-risk MM with t(4;14), del(17p), and R-ISS stage III disease. He had severe renal dysfunction (eGFR 15 mL/min/1.73 m^2^), anemia, and multiple vertebral compression fractures. His ECOG performance status was 3, and he was non-ambulatory because of severe pain. His IMWG frailty score was 4 (age, 1; ADL, 1; IADL, 1; comorbidity, 1), classifying him as frail.

Based on the 1st M (Myeloma Status/Matters Most), the team defined the immediate goal as rapid cytoreduction with renal preservation. In the 2nd M (Medications/Modalities), carfilzomib, lenalidomide, and dexamethasone (KRd) was considered based on biological risk. In the 3rd and 4th Ms (Mobility/Multi-morbidity and Mind [Mental]/Support Members), feasibility assessment identified key constraints—non-ambulatory status, limited cardiac reserve, and severe renal impairment. A safer induction regimen (daratumumab, bortezomib, and dexamethasone [D-Vd]) was therefore selected, with planned intensification to daratumumab, bortezomib, lenalidomide, and dexamethasone (D-VRd) once renal function improved and mobility recovered.

Framework insight: This case illustrates the adaptive, iterative logic of the 5M-OMM. A biologically appropriate regimen was first identified, then revised to account for functional and organ constraints, and re-intensified as feasibility improved. Without structured reassessment, a more intensive regimen might have been initiated despite borderline cardiac and renal function, increasing the risk of toxicity and treatment discontinuation. The framework supported stepwise intensification while maintaining feasibility, toward durable disease control.

## 4. Discussion

Treatment planning for older adults with MM requires balancing disease biology with functional and psychosocial factors and often warrants iterative adjustment as clinical status evolves.^20,40^ Instruments such as the IMWG frailty score and CGA provide valuable insights,^7,35,36^ but they were not designed to integrate information across domains or to support structured multidisciplinary discussion.^17,41^ Consequently, a disconnect may arise between biological assessment and real-world feasibility, contributing to variability in care delivery and, at times, underuse of appropriate therapies.^18^ To address this gap, we developed the 5M-OMM as a structured, sequential approach that integrates biological, functional, and psychosocial information into a cohesive framework (Table 2 summarizes key differences from existing tools).

**Table 2.** Comparison of Existing Tools and the 5M Framework for Older Adults with Multiple Myeloma (5M-OMM)

**Feature**	**IMWG frailty score**	**CGA**	**5M-OMM**
Primary purpose	Risk stratification	Comprehensive geriatric evaluation	Structured, stepwise decision-making
Domains assessed	Age; ADL/IADL; comorbidity (CCI)	Functional, cognitive, psychological, social, comorbidity, medication, and nutritional domains	Disease biology; patient priorities; therapeutic options; functional status and comorbidity burden; psychosocial context
Integration of patient values	Indirect	Present but not explicit	Explicit from the outset (via “Matters Most”)
Multidisciplinary relevance	Limited	High	High (each domain linked to team roles)
Framework nature	Static	Detailed but complex	Sequential, adaptable, and iterative (cyclical reassessment)

Abbreviations: IMWG, International Myeloma Working Group; ADL, activities of daily living; IADL, instrumental activities of daily living; CCI, Charlson Comorbidity Index; CGA, comprehensive geriatric assessment.

A central feature of the 5M-OMM is its explicit sequencing, which begins with the “biological imperative”—defining disease risk (the 1st M: Myeloma Status). Characterizing cytogenetic risk, disease aggressiveness, and organ-threatening involvement does not in itself mandate intensive therapy; rather, it delineates the therapeutic envelope—the range of medically appropriate options within which patient-centered choices can be made. For many patients of advanced age or marked frailty, disease stability or partial remission may be the most appropriate goal. Without defining biological risk at the outset, however, clinicians may select regimens that are either unnecessarily intensive or insufficient for adequate disease control. By placing Myeloma Status first, therapeutic ambition remains proportional to disease risk and clinical need, helping to avoid both overtreatment and undertreatment, as illustrated in Case 1. In contrast, approaches that begin with functional assessment may, at times, bias decisions toward more conservative strategies.^42,43^

Equally important, the 5M-OMM incorporates patient-defined goals and priorities from the outset of treatment planning (the 1st M: Matters Most). Case 2 illustrates how early clarification of what matters most—such as preserving independence or maintaining caregiving responsibilities—can directly inform regimen selection and treatment logistics. Integrating patient goals alongside disease biology makes shared decision-making more actionable and supports plans that are both medically appropriate and goal-concordant—a balance that can be difficult to achieve without a structured process.^44^ With disease biology and patient priorities established, the framework then turns to evidence-based selection of a provisional regimen.

Building on these principles, the 2nd M (Medications/Modalities) guides evidence-based selection among therapeutic options by weighing expected efficacy, toxicity, regimen complexity, and overall treatment burden. This step specifies a provisional regimen and clarifies the key trade-offs to be examined during subsequent feasibility assessment. By assessing feasibility only after a provisional regimen is specified, the 5M-OMM helps avoid premature de-escalation based on early impressions of frailty and enables deliberate, stepwise refinement.

Practical feasibility is then assessed through the 3rd M (Mobility/Multi-morbidity) and the 4th M (Mind [Mental]/Support Members). These domains capture functional reserve, comorbidity burden, cognition, psychological well-being, and social resources, thereby informing what can be delivered safely and sustained over time. If feasibility remains limited despite optimized supportive care, the team returns to the 2nd M to reconsider options and specify a revised regimen. Frailty is heterogeneous and cumulative; its clinical implications may differ when vulnerability is driven primarily by chronological age versus functional impairment (ADL/IADL) and comorbidity burden.^45^ Moreover, higher frailty scores (e.g., an IMWG frailty score ≥3) may indicate a narrower therapeutic margin for treatment intensity and tolerability, as suggested by subgroup analyses within the IMWG frailty score.^46^ As illustrated in Cases 1 and 3, multidisciplinary input—from hematologists, pharmacists, rehabilitation therapists, nurses, and social workers—clarifies roles and helps translate frailty assessment into actionable constraints and intervention targets.^42^ This team-based approach may also promote shared ownership, reduce fragmentation, and strengthen continuity of care.^47^

Finally, the 5M-OMM incorporates the 5th M (Menu/Motion) to translate these clinical, functional, and psychosocial considerations into an individualized treatment plan and its practical implementation over time, while formalizing iterative reassessment. This step specifies the treatment “menu” (dose, schedule, route, supportive measures, and monitoring) and coordinates the “motion” required to sustain treatment delivery (transportation, caregiver coordination, rehabilitation, adherence support, and symptom surveillance). The framework includes a feedback loop to enable timely adjustment as feasibility changes. Case 3 exemplifies this approach: treatment intensity was de-escalated in response to impaired mobility and renal dysfunction, with re-intensification planned once the patient’s condition improved. This cyclical decision-making reflects the non-linear trajectories often observed in older adults with MM.^29^ Frailty can also evolve during therapy; in the MAIA and CEPHEUS trials, frailty classification changed in 26–34% of patients over four years of follow-up.^48^ Embedding reassessment within the framework therefore supports both adaptive and sustainable treatment delivery across the disease course.

Several limitations warrant acknowledgment. The 5M-OMM is a conceptual framework supported by illustrative cases rather than a validated clinical tool, and its implementation may vary across institutions owing to differences in resources, team composition, and workflow. Accordingly, it is intended to provide structure—not to replace existing tools or clinical judgment—and should not be applied rigidly. Future studies should evaluate feasibility, reproducibility, and clinical impact across diverse practice settings.

In summary, the 5M-OMM integrates disease biology, patient priorities, frailty-related constraints, and multidisciplinary expertise within a sequential, adaptable process. By addressing not only which treatments are selected but also how they are delivered, it supports feasible, goal-concordant care for older adults with MM.

## 5. Conclusion

The 5M-OMM is an adaptable framework for guiding treatment decisions in older adults with MM. By integrating disease biology, patient priorities, functional status, and multidisciplinary input through an explicit sequence, it complements existing geriatric oncology tools and supports feasible, goal-concordant care across diverse clinical settings. Rather than treating biological imperatives and practical feasibility as competing considerations, the 5M-OMM frames them as interdependent elements of personalized treatment planning.

### Competing Interests

The author declares no relevant financial or non-financial interests to disclose.

### Ethics Approval

Not applicable. This study did not involve human participants or animals.

### Consent to Participate

Not applicable.

### Consent to Publish

Not applicable.

### Author Contributions

Conceptualization: Atsushi Isoda (Lead). Writing – original draft: Atsushi Isoda (Lead). Writing – review & editing: Atsushi Isoda (Lead).

## Supporting information

Supplementary Table S1

Supplementary Appendix

## Data Availability

Not applicable. No datasets were generated or analyzed during the current study.
