# Supplementary material for: Integrating biology, frailty, and patient values: The 5M framework for older adults with multiple myeloma
Source: Clin Hematol Int. 2026 Nov 3;8(3):77–86. doi: 10.46989/001c.171785 (PMC13738971; doi:10.46989/001c.171785)
Supplement: Supplementary file 1 [file chi_2026_8_3_171785_369853.docx]

**Supplementary Table S1. Detailed Assessment Items and Recommended Measurement Tools for Each Domain of the 5M Framework for Older Adults with Multiple Myeloma (5M-OMM)**

| **Domain** | **Key Assessment Items** | **Example Measures / Tools** |
| --- | --- | --- |
| 1st M: Myeloma Status/Matters Most | Disease-related assessment: disease biology and risk, disease aggressiveness, end-organ involvement, symptom burden, and prior treatment exposure/refractoriness.  What matters: patient priorities, goals of care, treatment trade-offs, and advance care planning | ISS/R-ISS (or R2-ISS, if used); high-risk cytogenetic abnormalities (e.g., del(17p), t(4;14), t(14;16), gain(1q)); CRAB features/organ involvement; pain assessment; prior lines of therapy and refractory status; patient-stated goals; goals-of-care discussion; ACP documentation (as appropriate) |
| 2nd M: Medications/Modalities | Evidence-based treatment options; treatment eligibility; toxicity risk; regimen complexity; feasibility of delivery; drug–drug interactions; and need for dose adjustment and supportive prophylaxis | IMiD-, PI-, and anti-CD38-based regimens; advanced immunotherapy eligibility (e.g., CAR T-cell therapy, bispecific antibodies); oral vs. IV/SC administration; renal dose adjustment; neuropathy risk assessment; corticosteroid tolerability; VTE prophylaxis review; infection prophylaxis and supportive medications |
| 3rd M: Mobility/Multi-morbidity | Frailty and functional status; fall risk; comorbidity burden; organ reserve; nutrition; polypharmacy | IMWG frailty score (preferred disease-specific frailty tool); ADL/IADL; gait speed; SPPB or Timed Up and Go test; comorbidity review/CCI; eGFR; cardiac assessment (as indicated); pulmonary assessment (as indicated); MNA-SF; BMI and unintentional weight loss; medication review |
| 4th M: Mind (Mental)/Support Members | Mentation: cognition, depression/anxiety, delirium risk, and adherence capacity/self-management ability.  Support system: caregiver availability, social support, transportation, and financial/access barriers | Mini-Cog or MoCA; GDS or PHQ-9; delirium risk screening (when relevant); adherence/medication management review; caregiver assessment; social support/loneliness measures; transportation access; home care/visiting nursing availability |
| 5th M: Menu/Motion | GA-guided multidisciplinary care planning and implementation: individualized treatment plan, supportive care plan, role assignment, follow-up intensity, and triggers for reassessment and treatment modification | Final treatment plan; toxicity mitigation plan; bone health, infection prevention, and rehabilitation referrals; role assignment within the multidisciplinary care team (e.g., hematology, geriatrics, pharmacy, nursing, rehabilitation, and social work); visit schedule; monitoring plan; predefined triggers for reassessment or treatment modification |

Abbreviations: ISS, International Staging System; R-ISS, Revised International Staging System; R2-ISS, Second Revision of the International Staging System; CRAB, hypercalcemia, renal insufficiency, anemia, and bone disease; ACP, advance care planning; IMiD, immunomodulatory drug; PI, proteasome inhibitor; CAR, chimeric antigen receptor; IV, intravenous; SC, subcutaneous; VTE, venous thromboembolism; IMWG, International Myeloma Working Group; ADL, activities of daily living; IADL, instrumental activities of daily living; SPPB, Short Physical Performance Battery; CCI, Charlson Comorbidity Index; eGFR, estimated glomerular filtration rate; MNA-SF, Mini Nutritional Assessment–Short Form; BMI, body mass index; MoCA, Montreal Cognitive Assessment; GDS, Geriatric Depression Scale; PHQ-9, Patient Health Questionnaire-9; GA, geriatric assessment.フォームの終わり
