# Supplementary material for: Integrating biology, frailty, and patient values: The 5M framework for older adults with multiple myeloma
Source: Clin Hematol Int. 2026 Nov 3;8(3):77–86. doi: 10.46989/001c.171785 (PMC13738971; doi:10.46989/001c.171785)
Supplement: Supplementary file 2 [file chi_2026_8_3_171785_369854.docx]

**Supplementary Appendix: Detailed Case Descriptions and Application of the 5M Framework for Older Adults with Multiple Myeloma (5M-OMM)**

**Case 1. Biological imperative and treatment initiation**

**1st M: Myeloma Status/Matters Most**

An 83-year-old man presented with gradually worsening low back pain. Imaging revealed multiple lytic lesions and an acute vertebral compression fracture with functional decline (ECOG performance status 3). Bone marrow examination confirmed symptomatic multiple myeloma (MM) with a relatively indolent clinical course. Cytogenetic testing showed no high-risk abnormalities, consistent with standard-risk disease, and the Revised International Staging System (R-ISS) stage was II. The International Myeloma Working Group (IMWG) frailty score was 4 (age >80 years, 2; ADL, 1; IADL, 1; comorbidity burden 0 [Charlson Comorbidity Index, CCI 0]), classifying him as frail. The patient prioritized prolonging survival, controlling pain, and receiving treatment at home whenever possible.

**2nd M: Medications/Modalities**

Given the standard-risk biological profile, regimen selection emphasized options supported by robust efficacy data. Although daratumumab, bortezomib, lenalidomide, and dexamethasone (D-VRd) was considered, limited high-quality evidence in patients >80 years supported selection of daratumumab, lenalidomide, and dexamethasone (DRd) as the preferred regimen. DRd was favored to reduce the risk of peripheral neuropathy relative to bortezomib-containing triplets and to facilitate an outpatient-oriented approach consistent with patient priorities. Contingency options included VRd; daratumumab, bortezomib, melphalan, and prednisone (D-VMP); Vd; and Rd. Implementation emphasized cautious corticosteroid use with early tapering, renal-adjusted lenalidomide dosing as needed, and subcutaneous daratumumab with appropriate premedication. Supportive care included antiviral prophylaxis, venous thromboembolism prevention, vaccination review, and initiation of bone-modifying agents after dental evaluation. Visit frequency was individualized based on mobility and caregiver availability.

**3rd M: Mobility/Multi-morbidity**

The vertebral fracture did not require radiotherapy, and pain was controlled with opioids and nonpharmacologic measures. He was wheelchair dependent for transfers but motivated to engage in rehabilitation with the goal of returning home. Physical therapy focused on trunk stabilization, safe transfer training, and fall prevention. The only major comorbidity was type 2 diabetes, for which antihyperglycemic therapy was optimized during hospitalization with nutritional counseling. Diabetic neuropathy was noted, further supporting avoidance of bortezomib. Renal function and bone marrow reserve were preserved, allowing initiation of lenalidomide without upfront dose reduction.

**4th M: Mind (Mental)/Support Members**

Screening identified mild short-term memory impairment without dementia. Several missed doses raised concern for post-discharge adherence. The patient planned to live with his daughter, who could oversee medication administration and accompany him to clinic visits. Depression screening was negative. Given a history of hospitalization-associated delirium, cognition was monitored closely, particularly during corticosteroid therapy. Home care services and visiting nursing were arranged to support adherence and activities of daily living.

**5th M: Menu/Motion**

The multidisciplinary team integrated the first four Ms into the following plan:

1. Initiate DRd using subcutaneous daratumumab with cautious corticosteroid dosing.
2. Begin inpatient rehabilitation for pain control and functional recovery, transitioning to home-based rehabilitation after discharge.
3. Optimize diabetes management and nutrition during hospitalization.
4. Support adherence through caregiver supervision and nursing follow-up.
5. Initiate advance care planning (ACP) during hospitalization.

Flexibility was maintained to adjust the plan—including revisiting the 2nd M—if toxicity or practical barriers emerged. The overarching aim was sustained disease control while preserving quality of life at home.

In summary, this case illustrates how the 5M-OMM aligned treatment intensity with biological risk and patient priorities and improved feasibility through targeted functional and social supports.

**Case 2. Alignment of therapy with patient values**

**1st M: Myeloma Status/Matters Most**

A 74-year-old woman was diagnosed with MM five years earlier and treated with six cycles of VRd, followed by lenalidomide maintenance. Over the preceding three months, she developed a gradual rise in M-protein levels and mild anemia without new bone lesions. Cytogenetic testing showed t(11;14), and relapse staging was R-ISS II, with no 1q gain or extramedullary disease. Her IMWG frailty score was 0, classifying her as fit. She prioritized maintaining caregiving responsibilities for her grandchild and minimizing hospitalizations, expressing a strong preference for outpatient management.

**2nd M: Medications/Modalities**

Relapse options with established efficacy were reviewed with attention to outpatient feasibility. Daratumumab, pomalidomide, and dexamethasone (DPd) was selected given its efficacy in lenalidomide-exposed disease, predominantly subcutaneous/oral administration, and compatibility with outpatient delivery. Alternative options included carfilzomib and dexamethasone (Kd), ixazomib, lenalidomide, and dexamethasone (IRd), and elotuzumab, pomalidomide, and dexamethasone (EPd). Although Kd was medically feasible in the absence of cardiac comorbidity, its infusion-intensive schedule was less compatible with her priorities. Scheduling was individualized to balance clinical monitoring with caregiver availability.

**3rd M: Mobility/Multi-morbidity**

Her ECOG performance status was 1, and she ambulated independently. Comorbidities included well-controlled hypertension and mild knee osteoarthritis without limitation in short-distance walking. Baseline renal and hepatic function were normal, and the absence of significant cytopenias supported initiation of pomalidomide without upfront dose reduction. A home-based exercise/physical therapy program was introduced to maintain mobility and reduce fall risk.

**4th M: Mind (Mental)/Support Members**

Cognitive and depression screening results were unremarkable. She had demonstrated excellent adherence over the prior five years. Her husband provided reliable support with clinic visits and symptom management. ACP had been initiated at diagnosis and was scheduled for review at relapse. Given stable social support and proven adherence, outpatient subcutaneous/oral therapy was considered highly feasible.

**5th M: Menu/Motion**

DPd was initiated in the outpatient setting, with hospitalization reserved for severe adverse events. Tolerance and feasibility were reassessed longitudinally, with readiness to revisit the 2nd M if regimen modification became necessary. The overarching aim was durable disease control while preserving quality of life and maintaining her daily activities, including caregiving.

In summary, this case illustrates how the 5M-OMM aligned relapse therapy with patient-defined priorities and outpatient feasibility while maintaining effective disease control.

**Case 3. Adaptive modification and longitudinal reassessment**

**1st M: Myeloma Status/Matters Most**

A 77-year-old man presented with worsening low back pain and generalized fatigue over two months. Diagnostic evaluation confirmed MM with high-risk cytogenetics (t(4;14) and del(17p), without 1q gain), and the R-ISS stage was III. Complications included renal impairment (creatinine 3.0 mg/dL; eGFR 15 mL/min/1.73 m²), anemia (hemoglobin 8.5 g/dL), and extensive lytic disease with vertebral fractures. His ECOG performance status was 3 with markedly impaired mobility; nevertheless, he remained motivated to pursue active therapy provided prolonged hospitalization could be avoided. The IMWG frailty score was 4 (age 76–80 years, 1; ADL, 1; IADL, 1; Charlson Comorbidity Index ≥2, 1), classifying him as frail.

**2nd M: Medications/Modalities**

Given the high-risk cytogenetics, rapid disease progression, and renal impairment, the therapeutic aim was prompt cytoreduction with renal preservation. Carfilzomib, lenalidomide, and dexamethasone (KRd) was considered because of its activity in high-risk myeloma. Alternative options included daratumumab, bortezomib, and dexamethasone (D-Vd) and bortezomib, cyclophosphamide, and dexamethasone (VCD). The initial plan was to assess feasibility for KRd induction, with readiness to transition to an alternative regimen if risks outweighed anticipated benefits.

**3rd M: Mobility/Multi-morbidity**

The patient was largely bedridden because of fracture-related pain. Although comorbidities were limited to hypertension, cardiac evaluation showed a mildly elevated BNP (196 pg/mL) and a left ventricular ejection fraction at the lower limit of normal (50%), raising concern for carfilzomib-associated cardiotoxicity. Severe renal dysfunction necessitated lenalidomide dose reduction, though partial reversibility was anticipated with treatment response. Bone marrow reserve was adequate to support bortezomib-based therapy. Taken together, KRd was judged to carry an unfavorable risk profile at presentation. The team therefore revisited the 2nd M and selected D-Vd as a safer initial regimen. Pain control incorporated pharmacologic and nonpharmacologic measures, and rehabilitation was initiated early to preserve functional capacity during treatment.

**4th M: Mind (Mental)/Support Members**

There was no evidence of cognitive impairment or depression, and medication history suggested strong adherence capacity. The patient’s elderly wife served as the primary caregiver but had limited physical reserve. A social worker coordinated with the local community support center to arrange visiting nursing, home-based rehabilitation, and transportation assistance.

**5th M: Menu/Motion**

D-Vd was initiated in the inpatient setting, prioritizing pain control and renal protection. Response and organ function were scheduled for reassessment after two cycles. If feasibility improved, escalation to daratumumab, bortezomib, lenalidomide, and dexamethasone (D-VRd) was planned. The care plan explicitly incorporated a re-loop strategy, allowing return to the 2nd M as clinical status and feasibility constraints evolved. The overarching aim was to balance aggressive disease control with safety while minimizing hospitalization and preserving quality of life.

In summary, this case illustrates the flexibility of the 5M-OMM, demonstrating adaptation from an initially intensive option to a safer regimen, with planned re-looping to the 2nd M as the patient’s condition evolved.
